# Supplementary material for: Splice-Junction-Based Mapping of Alternative Isoforms in the Human Proteome
Source: Cell Rep. Author manuscript; Available in PMC 2020 Jan 15. (PMC6961840; doi:10.1016/j.celrep.2019.11.026)
Supplement: 3 [file NIHMS1546469-supplement-3.zip › DF2/PXD0010154/Lung-347-A8MW92-GQDWIALVK.pdf]

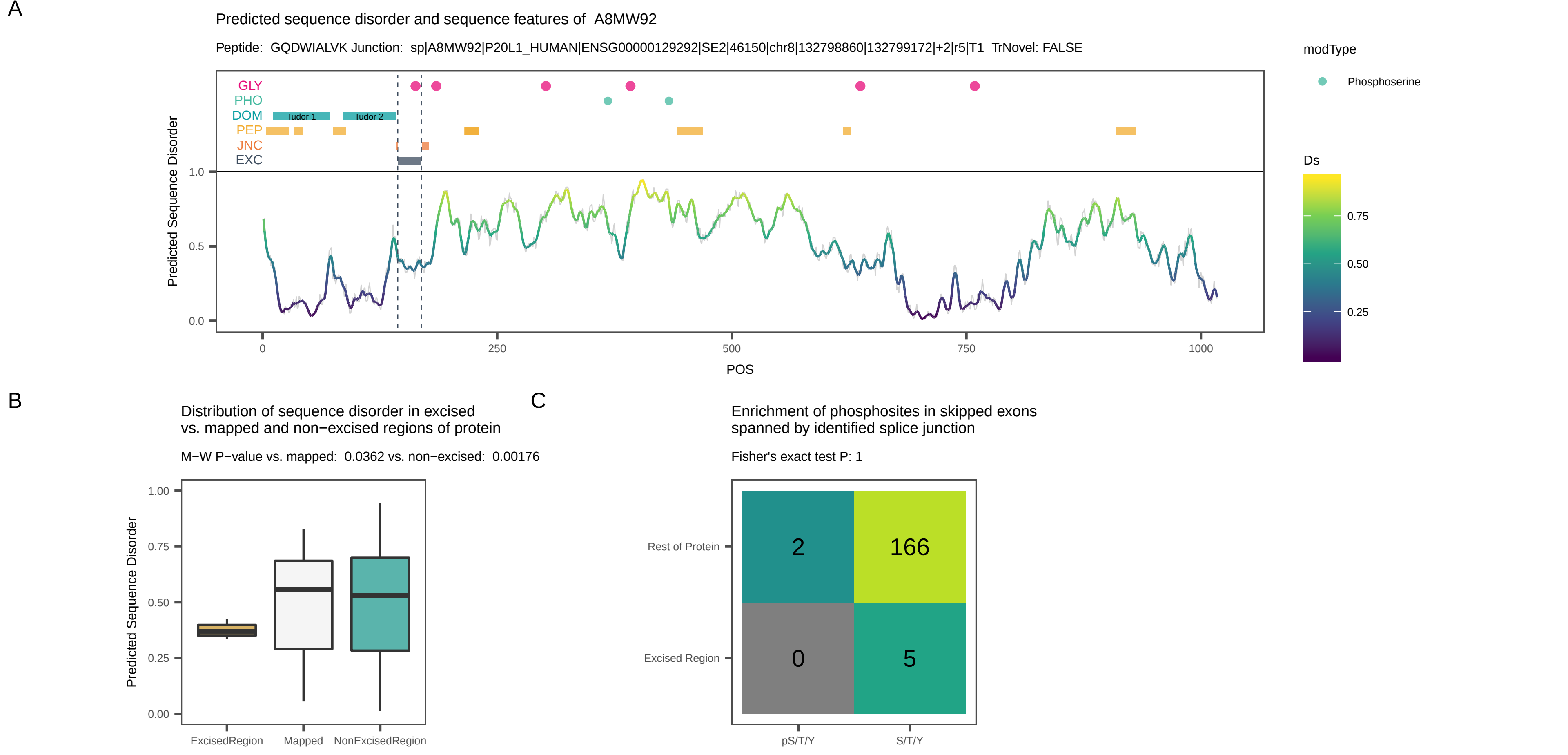

A
Predicted sequence disorder and sequence features of A8MW92
Peptide: GQDWIALVK Junction: sp|A8MW92|P20L1_HUMAN|ENSG00000129292|SE2|46150|chr8|132798860|132799172|+2|r5|T1 TrNovel: FALSE
GLY
PHO
DOM
PEP
JNC
EXC
Tudor 1
Tudor 2
Predicted Sequence Disorder
1.0
0.5
0.0
0
250
500
750
1000
POS
modType
Phosphoserine
Ds
0.75
0.50
0.25
B
Distribution of sequence disorder in excised vs. mapped and non−excised regions of protein
M−W P−value vs. mapped: 0.0362 vs. non−excised: 0.00176
Predicted Sequence Disorder
1.00
0.75
0.50
0.25
0.00
ExcisedRegion
Mapped
NonExcisedRegion
C
Enrichment of phosphosites in skipped exons spanned by identified splice junction
Fisher's exact test P: 1
Rest of Protein
Excised Region
2
166
0
5
pS/T/Y
S/T/Y
